# The Burden of Heart Failure and Its Impact on Mortality and Outcomes in Hospitalized Multiple Myeloma Patients: A Nationwide Study

**DOI:** 10.7759/cureus.47570

**Published:** 2023-10-24

**Authors:** Saad Javaid, Kelly Frasier, Ammad J Chaudhary, Olivia Del Castillo

**Affiliations:** 1 Internal Medicine, Wyckoff Heights Medical Center, New York, USA; 2 Internal Medicine, Nuvance Health, Poughkeepsie, USA; 3 Internal Medicine, Henry Ford Health System, Detroit, USA; 4 Internal Medicine, Community College, Beacon, USA

**Keywords:** secondary outcomes, healthcare utilization, in-hospital mortality, multiple myeloma, heart failure

## Abstract

Background

Multiple myeloma is a hematologic malignancy characterized by its association with a range of cardiovascular comorbidities, most notably heart failure. Our study aims to investigate the impact of heart failure on individuals who are hospitalized for multiple myeloma.

Methods

In this retrospective cohort study, we assembled a cohort of patients diagnosed with multiple myeloma from the National Inpatient Sample (NIS) data from 2019 to 2020. Within this study population, patients were classified according to the presence or absence of heart failure as a secondary diagnosis, with further stratification into distinct groups such as heart failure with reduced ejection fraction (HFrEF) and heart failure with preserved ejection fraction (HFpEF). The primary outcome studied was inpatient mortality. Secondary outcomes were length of stay, total hospitalization charges, acute respiratory failure, acute kidney injury, intensive care unit (ICU) admission, and mechanical ventilation. Confounders were adjusted using multivariate regression analysis.

Results

Among the 38,735 patients admitted with multiple myeloma, 5.6% (2,195 patients) were diagnosed with HFpEF, while 3% (1,170 patients) had HFrEF. The mortality rate was significantly higher in HFpEF patients compared to HFrEF and non-heart failure individuals (aOR: 1.68, [CI: 1.17-2.43]; P = 0.005). Length of hospital stay did not differ between these two groups; however, total hospitalization charges were more significant in the presence of heart failure versus without heart failure (coefficient: 33597; CI: 1730-65463; P = 0.04; and coefficient: 26107; CI: 5414-46800; P = 0.01 for HFrEF and HFpEF, respectively). Similarly, a significant increase in the odds of acute respiratory failure, care at the ICU, and requirement for mechanical ventilation was observed in patients with both types of heart failure compared to those without heart failure.

Conclusion

HFpEF was associated with high mortality rates and greater incidence of acute kidney injury in multiple myeloma patients compared to those with HFrEF and non-heart failure counterparts. However, both heart failure subtypes were associated with heightened total hospitalization charges and the increased likelihood of encountering acute respiratory failure, admission to the ICU, and the utilization of mechanical ventilation compared to patients without heart failure.

## Introduction

Multiple myeloma is a spectrum of conditions affecting different organ systems of the body, leading to various manifestations such as renal dysfunction, anemia, bone lytic lesions, cardiovascular disorders, and electrolyte disorders. These hallmark manifestations collectively contribute to the disease's complexity and impact on patient health, ultimately leading to hospitalizations [1]. The primary cause of mortality in multiple myeloma stems from the complications arising from the disease, leading to multisystem failure. Heart failure (HF) constitutes a significant public health concern, manifesting a prevalence exceeding 5.8 million within the United States and surpassing 23 million globally, with an upward trajectory [2].

In a retrospective study, it was revealed that a substantial proportion of individuals diagnosed with multiple myeloma, approximately two-thirds, had preexisting cardiovascular conditions [3,4]. Furthermore, the study observed that within a follow-up period of six years, an alarming 70% of the study population developed cardiovascular disease. Among those, heart failure was the significant adverse effect, and our study is focused on HF as the principal focal point. HF might affect multiple myeloma patients in numerous ways. Individuals with HF experience a greater number of additional medical conditions compared to those without HF. Moreover, it is pertinent to note that HF exhibits certain overlapping complications with multiple myeloma, particularly concerning renal and cardiovascular manifestations. This notable correlation with comorbidities may potentially elevate the risk of both mortality and morbidity for HF patients. HF originates from diverse causes, ultimately leading to two primary subtypes: heart failure with preserved ejection fraction (HFpEF) and heart failure with reduced ejection fraction (HFrEF). Prior research in the literature has explored the effects of different treatment approaches for multiple myeloma on the occurrence and consequences of HF within this patient cohort [5]. However, it is noteworthy that no previous study has specifically investigated HF as an independent prognostic factor for mortality and adverse hospital outcomes in individuals with multiple myeloma.

Our research endeavor seeks to assess the influence of HF on patients with multiple myeloma, focusing on their mortality rates and various adverse hospital outcomes, such as resource utilization, length of stay, acute respiratory failures, and intensive care unit (ICU) admissions. Moreover, we aim to examine how each subtype of HF affects this particular patient group differently. By gaining insights into these factors, our study aims to enhance survival and hospital outcomes for this specific subpopulation by effectively addressing and managing the underlying HF subtype.

## Materials and methods

Data source

This retrospective study sought to gain insights by analyzing extensive hospitalization data obtained from the Health Care Utilization Project-National Inpatient Sample (HCUP-NIS). The HCUP-NIS is a widely accessible database that compiles comprehensive information on hospital discharges from 47 states in the United States, including the District of Columbia [6]. Utilizing a well-structured sampling method involving close to one-fifth of all discharges from community hospitals across the country, excluding long-term acute care facilities and rehabilitation centers, HCUP-NIS provides an accurate representation of over 97% of the entire US population. The utilization of this extensive dataset provided us with the opportunity to conduct an in-depth analysis of a wide range of healthcare outcomes and trends within our target population. Given the de-identified and publicly available nature of the HCUP-NIS data, this study was considered exempt by the Institutional Review Board. This classification also demonstrates compliance with ethical standards and regulations governing human subjects' research.

Study design and population

In our study, we extensively analyzed a large sample of hospital discharges spanning from January 1, 2019, to December 31, 2020, and included a total of 38,735 cases that met our inclusion criteria. Inclusion criteria required that patients had to be at least 18 years old and should have been discharged with multiple myeloma as the primary diagnosis, with or without the secondary diagnosis of HF. Among this cohort, 4845 individuals presented with a secondary diagnosis of HF. Within this subgroup, 1170 patients exhibited HFrEF, while 2195 displayed HFpEF. An additional 1480 patients were diagnosed with unspecified HF and HF with both systolic and diastolic dysfunction; however, these cases were excluded from our study due to the non-specific nature of the cardiac dysfunction. We utilized internationally recognized coding systems, including ICD-10-CM and PCS classifications, to identify our diagnosis [7]. A separate supplementary table is provided with all the relevant ICD-10 codes for the conditions studied in the current study.

Outcomes

The primary outcome was inpatient mortality in multiple myeloma patients, which we studied separately for HFrEF and HFpEF compared to those with no heart failure. The secondary endpoints encompassed the following: (1) assessments of health care resource utilization, which included length of hospital stays (LOS), and total hospitalization charges, which were measured by multiplying the total charge and cost-to-charge ratio per hospitalization event; (2) disease severity, including acute kidney injury, acute respiratory failure, mechanical ventilation, and admission to the ICU. Distinct ICD-10 codes were assigned to each of the aforementioned conditions. The potential confounding factors that were considered and subsequently adjusted for included age, race, gender, median income, Carlson comorbidity index, hospital region, rural or urban location classification, hospital size categorized by bed count (small, medium, and large), and the teaching status of the hospital (teaching vs nonteaching).

Statistical analysis

Data analysis and extraction were conducted utilizing the Stata/BE 17.0 statistical software (StataCorp., College Station, Texas). In order to compare continuous variables such as age, LOS, and hospitalization cost, the student's t-test was employed for the statistical analysis. The results for these variables were reported in terms of their mean value and standard deviation. By utilizing this methodological approach, we were able to assess the differences between the various continuous factors under investigation. The Pearson chi-square test was employed to analyze categorical variables, such as in-hospital mortality. The results for these variables were expressed as weighted frequency percentages to provide a clearer understanding of their distribution and impact.

Furthermore, in order to establish the association between HF and hospital outcomes, we performed a statistical analysis aimed at determining the odds ratio of in-hospital mortality, the incidence rate ratio for LOS, and the cost ratio when comparing patients with HF to those without this underlying condition. This allowed us to evaluate the impact of HF on various aspects, such as mortality rates, duration of hospitalization, and associated costs, compared to individuals without HF.

## Results

This study employed an extensive dataset comprising 38,735 hospitalizations with multiple myeloma. Among these patients, 5.6% (2195 patients) exhibited a concomitant diagnosis of HFpEF, while 3% (1170 patients) presented a concurrent diagnosis of HFrEF. The mean age of patients with heart failure was significantly higher as compared to those with no heart failure (72.76 ± 10.64 and 70.19 ± 9.94 in HFpEF and HFrEF, respectively, vs 65 ± 11.2 years, P < 0.001). The Charlson comorbidity index was ≥3 in all patients with HF as compared to those with no HF (100% vs 58.29%, P < 0.001) [8]. In the context of racial demographics, individuals diagnosed with multiple myeloma (MM) and HFpEF demonstrated a higher prevalence of white ethnicity in contrast to those with HFrEF (64.9% vs 59.65%) and individuals without heart failure (61.76%). Conversely, patients with HFrEF exhibited a heightened likelihood of being of black ethnicity when compared to those with HFpEF (32.89% vs 27.16%) and the non-heart failure cohort (23.81%). The Medicare cohort demonstrated a greater prevalence of patients diagnosed with HFpEF (76.12%), whereas the private insurance group exhibited an increased proportion of non-heart failure patients (37.02%), followed subsequently by those with HFrEF (20.89%). HFpEF patients exhibited a higher prevalence of hypertension (97.72%), diabetes mellitus (24.15%), hyperlipidemia (41.23%), and obesity (16.4%) when compared to their HFrEF counterparts. Conversely, the HFrEF group had a higher proportion of patients with fluid and electrolyte disorders (64.1%), coronary artery disease (36.75%), and atrial fibrillation (32.91%). The detailed baseline characteristics and comorbidities of the two patient groups are presented in Table 1.

**Table 1:** Demographic data and comparison of baseline characteristics of multiple myeloma patients with and without heart failure HFrEF: Heart failure with reduced ejection fraction; HFpEF: Heart failure with preserved ejection fraction; SD: Standard deviation.

No. of patients	MM without HF	MM with HFrEF	P-value	MM without HF	MM with HFpEF	P-value
Patient characteristics	33890	1170		33890	2195	
*Gender (%)*	* *	* *	*P = 0.003*	* *	* *	*P = 0.023*
Male	19019 (56.12)	770 (65.81)		19019 (56.12)	1120 (51.03)	
Female	14871 (43.88)	400 (34.19)		14871 (43.88)	1075 (48.97)	
*Age*	* *	* *	*P < 0.001*	* *	* *	*P < 0.001*
Mean age (SD)	65 (11.12)	70.19 (9.94)		65 (11.12)	72.76 (10.62)	
*Age distribution (%)*	* *	* *	*P < 0.001*	* *	* *	*P < 0.001*
18-35	261 (0.77)	0		261 (0.77)	0	
36-45	1312 (3.87)	10 (0.85)		1312 (3.87)	15 (0.68)	
46-64	14356 (42.36)	340 (29.06)		14356 (42.36)	455 (20.73)	
>65	17965 (53.01)	820 (70.09)		17965 (53.01)	1725 (78.59)	
*Race (%)*	* *	* *	*P = 0.004*	* *	* *	*P = 0.003*
White	20930 (61.76)	698 (59.65)		20930 (61.76)	1425 (64.9)	
Black	8069 (23.81)	385 (32.89)		8069 (23.81)	596 (27.16)	
Hispanic	3592 (10.6)	72 (6.14)		3592 (10.6)	142 (6.49)	
Other	1298 (3.83)	15 (1.32)		1298 (3.83)	32 (1.44)	
*Median household income national quartile for patient zip code (%)*	* *	* *	*P = 0.632*	* *	* *	*P = 0.135*
$1-$49,999	8737 (25.78)	354 (30.22)		8737 (25.78)	672 (30.61)	
$50,000-$64,999	8083 (23.85)	270 (23.11)		8083 (23.85)	477 (21.73)	
$65,000-$85,999	8635 (25.48)	276 (23.56)		8635 (25.48)	574 (26.17)	
>$86,000	8432 (24.88)	270 (23.11)		8432 (24.88)	472 (21.5)	
*Charlson comorbidity index (%)*	* *	* *	*P < 0.001*	* *	* *	*P < 0.001*
2	14136 (41.71)	0		14136 (41.71)	0 (0)	
3 or more	19754 (58.29)	1170 (100)		19754 (58.29)	2195 (100)	
*Insurance provider (%)*	* *	* *	*P < 0.001*	* *	* *	*P < 0.001*
Medicare	17619 (51.99)	853 (72.89)		17619 (51.99)	1671 (76.12)	
Medicaid	3033 (8.95)	57 (4.89)		3033 (8.95)	109 (4.96)	
Private	12549 (37.03)	244 (20.89)		12549 (37.03)	389 (17.73)	
Uninsured	688 (2.03)	16 (1.33)		688 (2.03)	26 (1.18)	
*Comorbidities (%)*	* *	* *	* *	* *		
Hypertension	13820 (40.78)	35 (2.99)	P < 0.001	13820 (40.78)	2145 (97.72)	P < 0.001
Diabetes mellitus	5741 (16.94)	225 (19.23)	P = 0.516	5741 (16.94)	530 (24.15)	P < 0.001
Fluid and electrolyte disorders	18419 (54.35)	750 (64.1)	P = 0.009	18419 (54.35)	1380 (62.87)	P = 0.002
*Chronic kidney disease*						
Stage 2	430 (1.27)	35 (2.99)	P = 0.034	430 (1.27)	40 (1.82)	P = 0.397
Stage 3	2704 (7.98)	220 (18.8)	P < 0.001	2704 (7.98)	480 (21.87)	P < 0.001
Stage 4	1179 (3.48)	70 (5.98)	P = 0.159	1179 (3.48)	250 (11.39)	P < 0.001
Stage 5	200 (0.59)	0	P = 0.235	200 (0.59)	30 (1.37)	P = 0.034
ESRD	1681 (4.96)	135 (11.54)	P <0.001	1681 (4.96)	180 (8.2)	P = 0.015
Unspecified	2525 (7.45)	120 (10.26)	P = 0.165	2525 (7.45)	200 (9.11)	P = 0.329
Hyperlipidemia (HLD)	9459 (27.91)	435 (37.18)	P = 0.007	9459 (27.91)	905 (41.23)	P < 0.001
Coronary artery disease (CAD)	3640 (10.74)	430 (36.75)	P < 0.001	3640 (10.74)	680 (30.98)	P < 0.001
Atrial fibrillation	3409 (10.06)	385 (32.91)	P < 0.001	3409 (10.06)	615 (28.02)	P < 0.001
Obesity	3450 (10.18)	110 (9.4)	P = 0.5820	3450 (10.18)	360 (16.4)	P < 0.001
*Discharge disposition (%)*	* *	* *	*P < 0.001*	* *	* *	*P < 0.001*
Home	24685 (72.84)	603 (51.55)		24685 (72.84)	1132 (51.56)	
Home with home health	8069 (23.81)	494 (42.24)		8069 (23.81)	995 (45.33)	
Skilled nursing facility	918 (2.71)	44 (3.73)		918 (2.71)	61 (2.77)	
Against medical advice	217 (0.64)	29 (2.48)		217 (0.64)	8 (0.35)	
Hospital characteristics (%)	* *	* *				
*Bed size of hospital (STRATA)*	* *	* *	*P = 0.209*	* *	* *	*P = 0.471*
Small	4714 (13.91)	145 (12.39)		4714 (13.91)	290 (13.21)	
Medium	6670 (19.68)	290 (24.79)		6670 (19.68)	495 (22.55)	
Large	22506 (66.41)	735 (62.82)		22506 (66.41)	1410 (64.24)	
*Hospital location*	* *	* *	*P = 0.736*	* *	* *	*P = 0.193*
Rural	1000 (2.95)	40 (3.42)		1000 (2.95)	90 (4.1)	
Urban	32890 (97.05)	1130 (96.58)		32890 (97.05)	2105 (95.9)	
*Hospital teaching status*	* *	* *	*P = 0.122*	* *	* *	*P = 0.004*
Nonteaching hospital	3789 (11.18)	175 (14.96)		3789 (11.18)	355 (16.17)	
Teaching hospital	30101 (88.82)	995 (85.04)		30101 (88.82)	1840 (83.83)	
*Region of hospital*	* *	* *	*P = 0.082*	* *	* *	*P = 0.825*
Northeast	7435 (21.94)	290 (24.79)		7435 (21.94)	465 (21.18)	
Midwest	7364 (21.73)	295 (25.21)		7364 (21.73)	520 (23.69)	
South	12882 (38.01)	445 (38.03)		12882 (38.01)	845 (38.5)	
West	6209 (18.32)	140 (11.97)		6209 (18.32)	365 (16.63)	

Table 2 displays the adjusted odds ratio of in-hospital mortality between the two groups. After accounting for potential confounding variables, HFpEF among multiple myeloma patients demonstrated a statistically significant association with high mortality (aOR: 1.68, CI: 1.17-2.43, P = 0.005). However, no discernible difference in mortality was observed between individuals with HFrEF and those without HF (aOR: 1.26, CI: 0.75-2.12, P = 0.38).

**Table 2:** Adjusted odds ratio of mortality for hospitalized multiple myeloma patients with and without heart failure CI: Confidence interval; HFrEF: Heart failure with reduced ejection fraction; HFpEF: Heart failure with preserved ejection fraction.

Mortality in multiple myeloma patients with HF in comparison with no HF						
	Multiple myeloma with HFrEF			Multiple myeloma with HFpEF		
Logistic regression model	Odds ratio	95% CI	P-value	Odds ratio	95% CI	P-value
*Mortality*						
Unadjusted odds ratio	2.2	(1.41-3.43)	P = 0.001	2.53	(1.82-3.51)	P < 0.001
Adjusted odds ratio	1.26	(0.75-2.12)	P = 0.38	1.68	(1.17-2.43)	P = 0.005

There was no difference in the LOS in patients with HF versus those without HF (coefficient: 2.16; CI: 0.04-4.29; P = 0.004 and coefficient: 1.48; CI: 0.28-2.69; P = 0.02 for HFrEF and HFpEF, respectively). Despite a p-value below 0.005, the confidence interval overlap tends to diminish the statistical significance of these findings. However, total hospitalization charges were significantly increased in patients with both HFrEF and HFpEF as compared to those with no heart failure (coefficient: 33597; CI: 1730-65463, P = 0.04 and coefficient: 26107; CI: 5414-46800; P = 0.01 for HFrEF and HFpEF, respectively, Table 3).

**Table 3:** Adjusted incidence rate ratio of length of stay and total charges in hospitalized multiple myeloma patients LOS: Length of stay; TOTCHG: Total charges; USD: United States Dollar; HFrEF: Heart failure with reduced ejection fraction; HFpEF: Heart failure with preserved ejection fraction.

Regression analysis for LOS and TOTCHG in multiple myeloma patients with HF in comparison with no HF						
	Multiple myeloma with HFrEF			Multiple myeloma with HFpEF		
Linear regression model	Coefficient	95% CI	P-value	Coefficient	95% CI	P-value
Length of hospitalization (days)						
Univariate regression	2.24	(0.17-4.32)	P = 0.03	0.7	(-0.45-1.86)	P = 0.23
Multivariate regression	2.16	(0.04-4.29)	P = 0.04	1.48	(0.28-2.69)	P = 0.02
Total hospital cost (USD)						
Univariate regression	32383	(-79-64845)	P = 0.05	12446	(-8972-33864)	P = 0.26
Multivariate regression	33597	(1730-65463)	P = 0.04	26107	(5414-46800)	P = 0.01

HFpEF was associated with a high likelihood of developing acute kidney injury (AKI) as compared to patients with HFrEF and non-HF patients (aOR: 1.71; CI: 1.35-2.18; P < 0.001). Both HFrEF and HFpEF were associated with higher odds of acute respiratory failure (aOR: 2.00; CI: 1.33-3.02; P = 0.001 vs aOR: 2.50; CI: 1.85-3.39; P = 0.001, respectively), mechanical ventilation (aOR: 1.20; CI: 1.18-3.24; P = 0.009 vs aOR: 2.07; CI: 1.40-3.07; P = 0.001 respectively), and ICU admission (aOR: 2.11; CI: 1.36-3.28; P = 0.001 vs aOR: 1.78; CI: 1.25-2.51; P = 0.001, respectively). However, no difference in the development of sepsis or cardiac arrest was noted between the two groups (Table 4).

**Table 4:** Comparison of various secondary outcomes in hospitalized multiple myeloma patients with and without heart failure OR: Odds ratio; aOR: Adjusted Odds ratio; HFrEF: Heart failure with reduced ejection fraction; HFpEF: Heart failure with preserved ejection fraction.

	MM with HFrEF				MM with HFpEF			
Secondary outcomes	Unadjusted OR (95% CI)	P-value	Adjusted OR (95% CI)	P-value	Unadjusted OR (95% CI)	P-value	Adjusted OR (95% CI)	P-value
Acute respiratory failure	3.24 (2.27-4.62)	P < 0.001	2.00 (1.33-3.02)	P = 0.001	3.63 (2.79-4.71)	P < 0.001	2.50 (1.85-3.39)	P < 0.001
Cardiac arrest	3.88 (1.51-9.97)	P = 0.005	1.70 (0.49- 5.92)	P = 0.406	1.55 (0.55-4.36)	P = 0.402	0.94 (0.33- 2.68)	P = 0.903
Acute kidney injury	2.03 (1.56-2.64)	P < 0.001	1.15(0.83-1.59)	P = 0.397	2.73 (2.24-3.32)	P < 0.001	1.71 (1.35-2.18)	P < 0.001
Mechanical ventilation	2.81 (1.81-4.38)	P < 0.001	1.20 (1.18- 3.24)	P = 0.009	2.69 (1.90-3.80)	P < 0.001	2.07 (1.40-3.07)	P < 0.001
Sepsis	1.45 (0.89-2.35)	P = 0.133	1.26 (0.76- 2.09)	P = 0.369	1.44 (1.00-2.04)	P = 0.044	1.39 (0.95-2.05	P = 0.092
ICU admission	2.88 (1.94-4.28)	P < 0.001	2.11 (1.36- 3.28)	P = 0.001	2.29 (1.68- 3.12)	P < 0.001	1.78 (1.25-2.51)	P = 0.001

## Discussion

It is widely known that patients with MM are associated with a greater risk of developing underlying cardiovascular comorbidities than the general population, which may adversely affect the quality of life and have various other implications in this patient population [9]. Nonetheless, a significant void persists within the current body of literature concerning a comprehensive understanding of the impact of HF on MM, particularly in terms of its correlation with the manifestation of adverse in-hospital outcomes and hospital mortality. Consequently, in our study, we sought to address this research gap by conducting an investigation that shed light on and elucidated the intricate association between HF and the occurrence of adverse in-hospital outcomes and hospital mortality among individuals with MM.

Our study demonstrated that HFpEF is an independent predictor of inpatient mortality among patients hospitalized with MM. In contrast, HFrEF did not exhibit a significant autonomous link to mortality in this patient group. Nevertheless, HFrEF and HFpEF contribute to a more severe disease trajectory, resulting in high overall hospitalization expenses, acute respiratory failure, and the need for ICU admission. Previous research has established a connection between HF and unfavorable outcomes in cancer patients. Nouhravesh et al. conducted a study to evaluate the impact of HF on mortality rates among cancer patients [10]. This investigation revealed a significant increase in overall mortality among cancer patients experiencing HF. Likewise, Merdji et al. explored the relationship between cardiogenic shock and mortality in the context of cancer patients, identifying a substantial rise in long-term mortality within this group [11]. However, what sets our study apart is its refined approach.

In contrast to the previously mentioned studies, we introduced a more detailed classification of heart failure by differentiating between HFrEF and HFpEF. This methodological refinement enabled a discrete examination of the impact on mortality associated with each HF subtype, thereby enhancing the precision and depth of our insights into the effects of HF on mortality in the context of cancer patients. While the exact causative factors behind these intriguing observations regarding the disparity in mortality between the two HF cohorts remain undetermined, several underlying pathophysiological mechanisms merit consideration as potential contributors to this phenomenon. A review of the literature shows that MM patients are inherently prone to the development of or may present as a late-life complication with restrictive cardiomyopathy and high-output cardiac failure [12,13]. This severe manifestation of MM, especially with underlying HFpEF, might contribute to elevated mortality rates within this patient cohort. As the NIS does not provide information about the sequence of events or the post-discharge outcomes or follow-ups, the validity of our study findings should be verified with further prospective studies. As a result, special attention should be directed toward patients with underlying HFpEF and concurrent MM as this coexistence may substantially impact the survival outcomes of multiple myeloma patients.

Our research results suggest no difference in the length of hospital stay between patients diagnosed with MM, regardless of whether they had concurrent HF. However, significant differences were noticed in terms of total hospitalization expenses. Both groups affected by HF incurred higher charges than those without this comorbidity. This could be attributed to the gravity of the illness and the heightened complexity of HF cases within this group, resulting in a higher frequency of admissions to intensive care units and more extensive medical attention. These findings demonstrate concurrence with prior research endeavors that have explored the influence of HF on various other medical conditions [14-17]. This adverse effect remained significant even after accounting for the confounding variables that exhibited an unequal distribution between the two groups. These variables included but were not limited to comorbidity burden, sex, age, race, insurance status, and hospital region.

Additionally, our study highlighted the substantially increased burden of other concomitant comorbidities among these patients, and there exists a significant association between MM and heart failure as both conditions exhibit overlapping complications. Prior studies have established a correlation between HF, MM, and renal injury as separate and independent variables [18,19]. In line with this existing body of knowledge, our study aimed to examine the association between AKI and the coexistence of these two conditions in a specific subpopulation of patients. Our study revealed that, among patients with HF, the incidence of AKI demonstrated a significant association with preserved ejection fraction compared to those with reduced ejection fraction. However, apart from this specific finding, all other adverse outcomes studied in our study exhibited comparable occurrences between these two distinct subcategories. This potential disparity in adverse event occurrence between these two subgroups will provide valuable insight, particularly in managing patients with concomitant HFpEF.

Although the incidence of respiratory complications alone in patients with MM is rare, only a few cases have been reported in the review of the literature [20]. The proposed mechanisms include infiltration of plasma cells in the lung parenchyma, amyloid deposition in the alveolar septum, and metastatic calcification of alveolar walls and blood vessels. However, the impact could become significant if it has concurrent heart failure as demonstrated in our study, where patients with simultaneous HF faced an elevated risk of encountering acute respiratory failure, necessitating mechanical ventilation and requiring admission to the ICU when compared to patients with no HF.

These results underscore the clinical significance of understanding the impact of HF in the context of multiple myeloma as it appears to play a substantial role in exacerbating adverse outcomes, particularly concerning respiratory complications and critical care requirements.

The study conducted by Jackson et al. emphasized the significance of early integration of palliative care for patients diagnosed with MM, taking into consideration the disease burden [21,22]. This supports and validates the idea proposed in our study that a specific subpopulation of patients, particularly those with multiple comorbidities, can achieve improved outcomes when measures are taken to control the disease burden and therapeutic approaches are employed to address the underlying conditions. Such interventions have the potential not only to enhance survival rates but also to reduce the incidence of adverse outcomes among hospitalized patients.

Despite its extensive coverage of in-hospital patient outcomes, the NIS database exhibits certain limitations regarding the information it provides. Even though the ICD-10 codes are associated with higher specificity and positive predictive value for the diagnosis and aim to enhance the accuracy of data representation, the inherent limitations of coding systems and the possibility of coding errors should be considered when interpreting the results. Notably, this database lacks baseline laboratory and imaging information data, precluding a comprehensive understanding of the patient's initial health status at admission. An important aspect to consider is that the NIS records hospitalization data on a per-admission basis rather than a per-person basis. Consequently, the same individual can be counted multiple times in cases admitted to different hospitals or experienced multiple hospital stays within the study period. Researchers should be mindful of these limitations when interpreting and generalizing the findings based on the NIS database.

## Conclusions

HFpEF is an independent predictor of mortality, while HFrEF was not associated with mortality in patients with MM. However, both categories of HF were associated with increased resource utilization and incidence of acute respiratory failure, ICU admission, and mechanical ventilation compared to patients with no HF. High disease burden in HF patients might explain the high mortality rate and increased association with adverse hospital outcomes. Diligent management of underlying HF is associated with improved survival and reduced adverse outcomes in MM patients.

## Appendices

**Table 5:** ICD-10 codes of diagnosis and procedure variables ICU: Intensive care unit; ESRD: End-stage renal disease; CKD: Chronic kidney disease.

	ICD-10 codes
Heart failure	I50.20, I50.21, I50.22, I50.23, I50.30, I50.31, I50.32, I50.33, I50.40, I50.41, I50.42, I50.43, I50.9
Multiple myeloma	C90.0, C90.00, C90.01, C90.02
Hypertension	I10, I15, I15.0, I15.1,I15.2, I15.8, I15.9
Diabetes mellitus	E10x, E11x
CKD stage 2-5, ESRD, CKD unspecified	N18.1, N18.2, N18.3, N18.4, N18.5, N18.6, N18.9
Fluid and electrolyte disorders	E87.0, E87.1, E87.20, E87.21, E87.22, E87.29, E87.3, E87.4, E87.5, E87.6, E87.70, E87.71, E87.79, E87.8, E83.52, E83.51
Acute respiratory failure	J96.0, J96.00, J96.01, J96.02
Cardiac arrest	I46.2,I46.8, I46.9
Mechanical ventilation	5A1935Z, 5A1945Z, 5A1955Z, 5A09357, 5A09457, 5A09557
ICU admission	5A1935Z, 5A1945Z, 5A1955Z, 5A09357, 5A09457, 5A09557, 3E030XZ, 3E033XZ, 3E040XZ, 3E043XZ, 3E050XZ, 3E053XZ, 3E060XZ, 3E063XZ, 5A1223Z, 03HC3DZ, 03HB3DZ, 04HL3DZ, 04HK3DZ, 06HN33Z, 06HM33Z, 05H633Z, 05H533Z, 05HM33Z, 05HN33Z
Sepsis	A02.1, A22.7, A26.7, A32.7, A40.0, A40.1, A40.3, A40.8, A40.9, A41.1, A41.2, A41.3, A41.4, A41.50, A41.51, A41.52, A41.53, A41.59, A41.81, A41.89, A41.9, A42.7, A54.86, B37.7, R65.20, R65.21
Acute kidney injury	N17.0, N17.1, N17.2, N17.8, N17.9
